# Recombinant DNA/MVA/ChAdV-63-elicited T cells specific for conserved regions of the HIV-1 proteome recognize HIV-1 infected cells and suppress HIV-1

**DOI:** 10.1186/1742-4690-9-S2-P259

**Published:** 2012-09-13

**Authors:** T Ahmed, N Borthwick, H Yang, G Hancock, L Yorke, U Ebrahimsa, A Rose, A Black, E Hayton, A McMichael, L Dorrell, T Hanke

**Affiliations:** 1 Oxford University, Oxford, UK

## Background

Currently, immunogenicity assessments of candidate HIV-1 vaccines in human clinical trials rely on the use of peptides for sensitization of target cells. However, employment of HIV-1-infected cells provides more informative and relevant in vitro readouts of HIV-1 recognition.

## Methods

A viral suppression assay (VSA) was developed to assess CD8+ T cell responses to infected autologous CD4+ targets using HIV-1 p24 levels as measured by flow cytometry and ELISA. In parallel, we also investigated live virus infected cells for re-stimulation of vaccine elicited T cells in an IFN-γ ELISpot assay.

## Results

HIV-1 infection kinetics is influenced by the multiplicity of infection (MOI) used to infect autologous CD4+ cells, with rapid kinetics observed at higher MOIs. For HIV-1 BaL optimal infection of autologous CD4+ cells was achieved at MOI of 0.05. In the VSA, HIV-1 BaL virus replication was shown to be sensitive to the chemokines MIP-1α and RANTES added in the absence of effector cells. Pre-activated effector cells indicated increased non-specific background suppression in healthy controls, which was reduced following a prolonged rest before co-culture with autologous CD4+ targets, but led to marked proliferation of the CD8+ T cell effectors. Preliminary investigations in vaccinees show that HIV-1 suppression mediated by CD8+ T cells can be detected in vitro following vaccination and that P24 ELISA has higher sensitivity than flow cytometry. As an alternative to the VSA, an IFN-γ ELISpot assay has also been optimized for the use of autologous HIV-1-infected CD4+ cells. Using both of the above assays, preliminary characterization of T cell responses induced in volunteers receiving pSG2.HIVconsv DNA, MVA.HIVconsv and ChAdV63.HIVconsv vaccines will be shown.

## Conclusion

Two assays employing HIV-1 infected target cells were standardised and employed to characterize responses elicited in participants of the HIV-1 vaccine trial HIVCORE002 in Oxford, UK.

